# Post-Treatment Imaging in Focal Therapy: Understanding TARGET and PI-FAB Scoring Systems

**DOI:** 10.3390/diagnostics15111328

**Published:** 2025-05-26

**Authors:** Haidy Megahed, Samuel Tremblay, Jason Koehler, Simon Han, Ahmed Hamimi, Aytekin Oto, Abhinav Sidana

**Affiliations:** 1 Section of Urology, Department of Surgery, University of Chicago, Chicago, IL 60637, USA; samuel.tremblay@uchicagomedicine.org (S.T.); abhinavsidana@uchicago.edu (A.S.); 2 College of Medicine, University of Cincinnati, Cincinnati, OH 45267, USA; koehlejn@mail.uc.edu; 3 Pritzker School of Medicine, University of Chicago, Chicago, IL 60637, USA; simon.han@uchicagomedicine.org; 4 Department of Radiology, University of Chicago, Chicago, IL 60637, USA; ahmed.hamimi@bsd.uchicago.edu (A.H.); aoto@bsd.uchicago.edu (A.O.)

**Keywords:** prostate cancer, focal therapy, MRI scoring, TARGET, PI-FAB

## Abstract

As the adoption of focal therapy (FT) for prostate cancer (PCa) grows, the demand for accurate post-treatment imaging to monitor outcomes and detect residual or recurrent cancer increases. Traditional diagnostic systems like the Prostate Imaging Reporting and Data System (PI-RADS) are ill-suited for post-FT evaluations due to treatment-induced tissue changes. MRI-based scoring systems specific for evaluation after FT have been developed to address these challenges and improve post-FT imaging accuracy by distinguishing benign alterations from recurrence. The currently developed scoring systems are Transatlantic Recommendations for Prostate Gland Evaluation with MRI after Focal Therapy (TARGET) and Prostate Imaging after Focal Ablation (PI-FAB). In this review, we describe and compare these two systems. These scoring systems standardize imaging assessments, enhance follow-up care, and support clinical decision-making. While promising, TARGET and PI-FAB require further large-scale validation to confirm their utility. Nevertheless, they represent critical advances in optimizing PCa management, particularly for patients undergoing FT, by improving diagnostic accuracy and guiding treatment decisions.

## 1. Introduction

Prostate cancer (PCa) is the most common non-cutaneous cancer among men worldwide, particularly in developed countries. There are approximately 1.46 million new cases of prostate cancer globally and about 396,792 deaths attributed to the disease every year. Higher incidence rates are observed in North America and Europe, where widespread screening and awareness contribute to earlier detection. However, mortality rates are higher in regions with limited access to diagnostic tools and treatment options. This highlights the importance of improved early detection and treatment strategies worldwide [1,2].

Depending on the stage and aggressiveness of the disease, approximately 70% to 90% of men with localized prostate cancer may survive for 10 years on watchful waiting or active surveillance without immediate treatment [2,3]. However, most with localized PCa undergo radical whole-gland therapy, such as radical prostatectomy or radiation therapy, which can cause significant iatrogenic morbidities. Active surveillance (AS) is increasingly used for low-risk cases, but some patients still prefer radical treatments due to multiple factors like high disease volume, anxiety, or family history. For intermediate-risk cases, radical treatments remain common despite the low mortality risk, raising concerns about potential overtreatment. On the other hand, AS carries risks, as nearly 20% of patients progress to higher-risk categories requiring whole-gland therapy. These extremes, AS versus whole-gland treatment, reflect a spectrum of care, with each option presenting distinct risks and benefits [4,5,6,7,8].

Prostate gland ablation for PCa might provide the option for a “middle” ground between AS and radical therapy by destroying prostate cancer in a minimally invasive or non-invasive fashion and thus limiting the morbidity [9,10,11,12,13,14,15,16]. FT for PCa was introduced in the early 2000s as a minimally invasive alternative to traditional treatments. It employs various energy sources, such as high-intensity focused ultrasound (HIFU), cryotherapy, laser ablation, and irreversible electroporation (IRE), to selectively target and destroy cancerous tissue within the prostate [9,10,11,12,13,14,15]. The main advantage of FT is its ability to effectively treat cancer while preserving the prostate structure and maintaining urinary and sexual function, resulting in fewer side effects than radical treatments [16,17,18,19,20,21]. Therefore, the demand for precise and reliable post-focal therapy imaging is increasing as the use of FT increases to effectively monitor treatment success and detect any residual or recurrent cancer [22,23,24].

Multiparametric magnetic resonance imaging (mpMRI) has played a major role in improving the detection, assessment, and guiding biopsy decisions and management of prostate cancer, aligning with the global push for more accurate diagnostic tools and tailored treatment options [25,26,27,28,29,30,31,32]. Historically, prostate cancer detection relied heavily on prostate-specific antigen (PSA) screening and systematic transrectal ultrasound (TRUS)-guided biopsies, which often led to the overdiagnosis of indolent cancers while missing clinically significant ones [33]. The limitations of TRUS-guided biopsies, particularly their low sensitivity and specificity, prompted the search for more reliable imaging modalities [34]. Studies have shown that TRUS biopsies miss up to 30–40% of clinically significant cancers, highlighting the urgent need for advanced imaging techniques [34,35].

The introduction of mpMRI has marked a turning point in prostate cancer imaging. Unlike conventional imaging techniques, mpMRI combines three key sequences: T2-weighted imaging (T2WI), diffusion-weighted imaging (DWI), and dynamic contrast-enhanced imaging (DCE), allowing for improved differentiation between benign and malignant lesions. This multimodal approach enables a more precise localization of clinically significant prostate cancer (csPCa) and has been instrumental in reducing unnecessary biopsies while improving the detection of high-grade tumors [28,29,30,31,32,33,34,35].

Beyond its diagnostic utility, mpMRI has played an increasingly vital role in active surveillance strategies, focal therapy planning, and post-treatment monitoring. The development of mpMRI has also enabled the accurate localization of lesions within the prostate, which has allowed for the development of focal therapy (FT). Also, mpMRI has proven essential for assessing treatment response, identifying residual or recurrent disease, and guiding targeted biopsies [36,37,38,39,40,41,42,43].

In the primary diagnostic setting, the Prostate Imaging Reporting and Data System (PI-RADS) is now widely used [44,45,46,47,48,49,50,51]. The standardization of mpMRI interpretation has been significantly enhanced by the introduction of PI-RADS, which provides a structured framework for evaluating prostate lesions [52]. However, PI-RADS is not suitable for post-focal therapy evaluation due to the tissue changes induced by treatment, such as scar formation, inflammation, and altered vascularization, which can obscure or mimic signs of recurrence [53,54,55,56,57]. As such, there is a need for specific guidance for MRI assessment following focal therapy to accurately distinguish between benign post-treatment changes and residual or recurrent cancer [12,58]. Following FT, treated areas often show scarring, inflammation, hematomas, or changes that can resemble residual disease [17,59,60,61]. Distinguishing between these benign post-treatment effects and active cancer tissue is a significant challenge. Advanced techniques, such as diffusion-weighted imaging (DWI) and dynamic contrast-enhanced MRI (DCE-MRI), improve differentiation but are not foolproof, and false positives or negatives remain a concern [22,62,63,64]. Additionally, accurate interpretation of MRI post-FT requires specialized training and expertise, as reading MRI for post-treatment monitoring is more complex than for initial cancer diagnosis. Radiologists must be familiar with post-ablation tissue changes to avoid misinterpretation [65,66].

At 12 months post-focal therapy, mpMRI findings vary depending on the energy source used. For example, HIFU typically results in a persistent hypointense area on T2WI with reduced or absent contrast enhancement on DCE, indicating fibrosis and loss of vascularity [67,68]. Cryotherapy often shows a fibrotic hypointense zone on T2WI, often with minimal enhancement on DCE, while retaining a low signal in all sequences due to dense scarring, making recurrence assessment challenging [58]. IRE lesions may normalize on T2WI over time, appearing as subtle signal alterations rather than distinct lesions, with persistent DCE enhancement, mainly peripherally, potentially suggesting recurrence [59]. Laser ablation and PDT also produce long-term fibrotic hypointense regions on T2WI with minimal enhancement [69]. It is important to note that post-treatment mpMRI findings can be influenced by the method used, the extent of tissue ablation, and the healing response, which can mimic cancer recurrence or benign tissue changes, making interpretation challenging.

Moreover, the lack of standardized imaging protocols is a challenge. Currently, there are no universally accepted protocols for post-FT MRI assessment. Variability in MRI techniques, field strengths, and image interpretation criteria between institutions can lead to inconsistent findings. Therefore, standardization in protocols would improve the reliability and comparability of MRI results post-FT [70].

The Prostate Imaging for Recurrence Reporting (PI-RR) system was introduced in 2021 as a framework specifically aimed at the MRI assessment of local recurrence after radiation therapy or radical prostatectomy; however, it does not fully address the unique imaging challenges that arise following FT [70,71,72,73]. Scoring systems, such as the Transatlantic Recommendations for Prostate Gland Evaluation with MRI after Focal Therapy (TARGET) and Prostate Imaging after Focal Ablation (PI-FAB), have been developed to fill this gap. These systems are specifically designed to account for the changes observed in MRI post-FT and can improve diagnostic accuracy, enhance follow-up care, and aid in clinical decision-making for patients undergoing FT [74,75,76].

Background, principles, and criteria of scoring in the TARGET system:

## 2. Background

TARGET is a 5-point scoring system introduced in 2022 as a standardized framework for assessing prostate MRI after FT. Recognizing the limitations of the existing PI-RADS for post-therapy imaging, TARGET was developed by an international panel of experts to address the unique imaging challenges that arise following FT specifically [53,77,78].

Since its introduction, TARGET has aimed to provide structured guidelines to differentiate benign post-treatment changes, such as scarring and inflammation, from residual or recurrent prostate cancer. By establishing specific scoring criteria that consider the effects of FT, TARGET helps radiologists accurately interpret MRI findings, minimizing diagnostic uncertainty and enhancing patient management decisions in the post-therapy setting. In the TARGET system, the score is calculated by integrating findings from key MRI sequences—T2-weighted imaging (T2WI), diffusion-weighted imaging (DWI), and dynamic contrast-enhanced MRI (DCE-MRI)—each of which contributes specific information about tissue characteristics, vascularity, and diffusion within prostate post-focal therapy [74,77].

### 2.1. Key TARGET Recommendations

Regarding MRI timing, it is recommended that an MRI be performed 12 months after FT to evaluate recurrence, with subsequent surveillance MRI scheduled annually if the patient has a negative MRI and normal PSA [77,79,80]. Full multiparametric MRI, including T2W, DWI, and DCE sequences, is mandatory. While an MRI can be conducted with either a 1.5 T or 3.0 T scanner, the 3.0 T scanner is preferred. An external, phased array coil is standard, with no requirement for an endorectal coil. All technical specifications for T1W, T2W, DWI, and DCE sequences should align with PI-RADS version 2.1 standards, with DCE serving as the primary sequence graded out of 3 [77].

**Figure 1:**
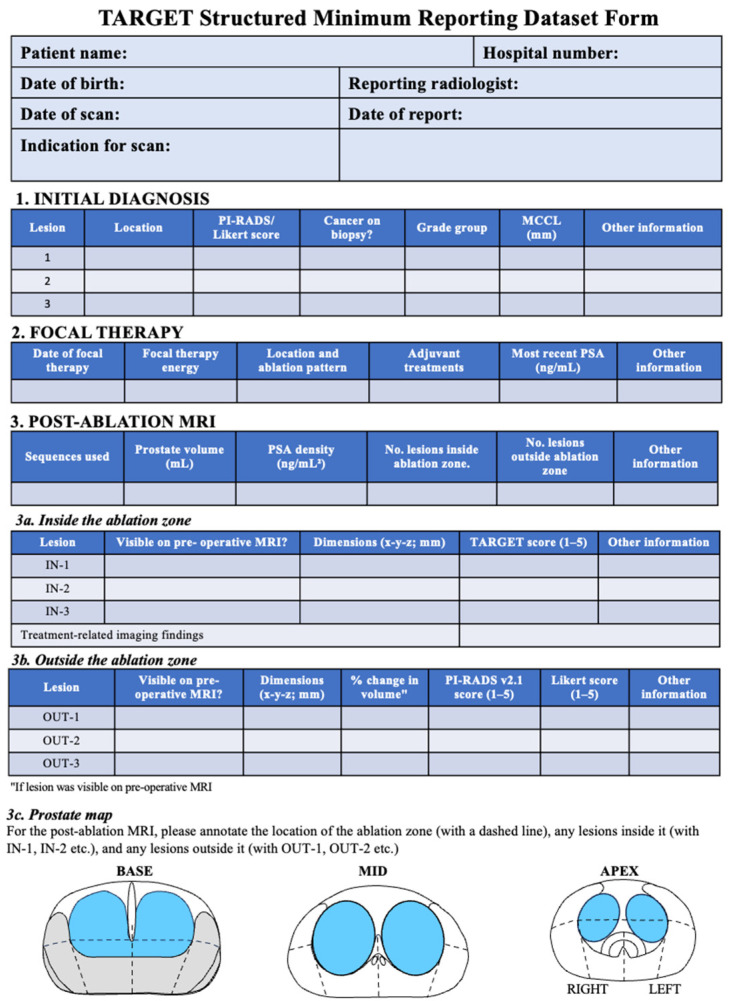
TARGET post-focal therapy MRI reporting data set. MCCL = maximum cancer core length; MRI = magnetic resonance imaging; PI-RADS = Prostate Imaging Reporting and Data System.

Comparison should be made with the most recent pre-therapy MRI, and readers should ideally review a minimum of 20 post-FT prostate MRIs annually. New readers should have their assessments double-checked by an experienced reader. MRI findings post-FT should be documented in a structured minimum reporting data set (Figure 1). For patients with a negative MRI and normal PSA levels at 12 months, a follow-up MRI is recommended unless a protocol biopsy is negative or omitted. Surveillance duration depends on individual cancer characteristics. In cases of inadequate imaging quality for T2W or DWI sequences pre-FT, these sequences should be redone preoperatively to ensure comparability [77,79,81]. Post-FT imaging may initially show treatment-related changes, such as hemorrhage, edema, or necrosis, while later scans may show fibrosis or fluid-filled cavities [77,82].

Lesions within the ablation zone are scored out of 5, whereas those outside the ablation zone are assessed using both PI-RADS version 2.1 and Likert scores. Clinical details, including therapy specifics, recent PSA values, and pre-therapy Gleason scores, should be available for MRI readers. Studies on MRI outcomes post-FT should meet reporting standards [77].

#### Calculating the TARGET Score

**Figure 2:**
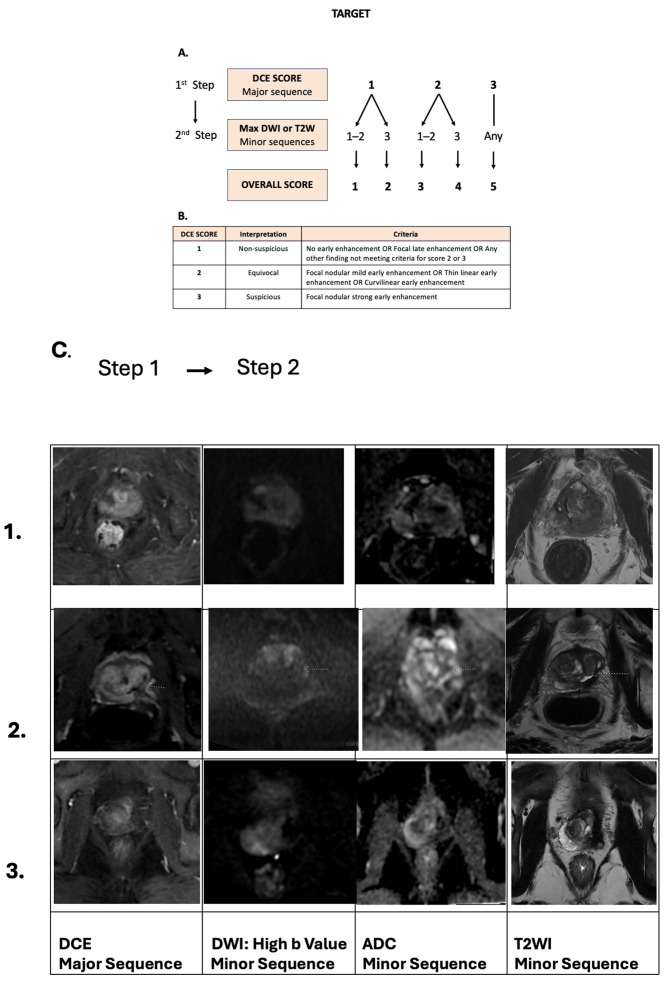
(**A**): DCE MRI is the principal imaging sequence using the 5-point TARGET scoring system, but T2WI and DWI are also used for minor, yet important, sequences. The above-mentioned algorithm describes the steps for reaching the TARGET score. Each sequence should be graded with a score of 1, 2, or 3. Overall, a TARGET score of 1 or 2 means very low to low suspicion of recurrence, a score of 3 is equivocal, a score of 4 is highly suspicious of recurrence, while a score of 5 is very highly suspicious. (**B**) Interpretation of DCE MRI scores. **C**: Example of MRI scans post-focal therapy: Row 1: MRI performed 12 months after right hemiablation with HIFU. At the anterior aspect near the apex, there is a focal lesion with restricted diffusion on ADC and high b-value images (DWI 3/3), with corresponding early enhancement (DCE 3/3). This would give an overall score of 5/5. Targeted biopsy was positive for recurrent cancer in 3/3 cores. Row 2: MRI performed 9 months after HIFU left hemiablation. At the left posterior aspect there is a focal lesion outlined by the dotted arrow in dynamic post contrast, DWI, ADC and T2WI respectively, there is an area with restricted diffusion on the high b-value image (DWI 3/3) and faint contrast enhancement (DCE 2/3). This would give an overall score of 4/5. Targeted biopsy of this lesion was positive for 2/3 cores sampled. Row 3: MRI performed 7 months after left-quadrant ablation with HIFU. There is no diffusion restriction (DWI 1/3) or contrast enhancement within the ablation zone (DCE 1/3), giving an overall score of 1/5. Template biopsies sampling the whole prostate were all negative. ADC = apparent diffusion coefficient; DCE = dynamic contrast-enhanced; DWI = diffusion-weighted imaging; HIFU = high-intensity focused ultrasound; T2W = T2-weighted.

TARGET, unlike PI-RADS v2.1, which is tailored to treatment-naïve glands, integrates a two-tiered scoring approach using major and minor MRI sequences [74,77] (Table 1) (Figure 2):

Major Sequence: Dynamic contrast-enhanced MRI (DCE-MRI) is the principal imaging sequence in TARGET, reflecting its strong capacity to distinguish benign from suspicious tissue changes post-therapy. DCE-MRI reveals vascular patterns that can help identify recurrence by highlighting differences between normal post-treatment changes and cancerous lesions.

Minor Sequences: Diffusion-weighted imaging (DWI) and T2-weighted imaging (T2WI) serve as complementary minor sequences. DWI helps identify restricted diffusion commonly associated with malignancy, while T2WI assesses tissue morphology and scarring.

In the TARGET scoring system, lesions within the ablation zone are evaluated on a 5-point scale that reflects the level of suspicion for recurrence. Each sequence—DCE-MRI, DWI, and T2WI—is rated on a scale of 1 to 3, with individual sequence scores indicating the following:

1 = Nonsuspicious

2 = Equivocal

3 = Suspicious

Combining Scores from All Sequences: Each sequence contributes to an overall assessment score, where DCE-MRI findings often play a decisive role, especially in ambiguous cases. Scoring typically uses a range from 1 to 5, with higher scores indicating a greater likelihood of recurrent cancer:

Score 1–2: Low likelihood of recurrence; findings are generally benign across all sequences, with no major red flags in T2WI, DWI, or DCE-MRI. Score 1 for very low suspicion and score 2 for low suspicion.

Score 3: Indeterminate; some suspicious features are present, but findings are inconclusive, often warranting additional imaging or close follow-up.

Score 4–5: High likelihood of recurrence; suspicious findings are consistent across multiple sequences, particularly with rapid uptake and washout on DCE-MRI, suggesting probable recurrent cancer. Score 4 for high suspicion and score 5 for very high suspicion.

Final Interpretation: The final TARGET score synthesizes the information gathered across sequences. Scores of 4 or 5 typically prompt further diagnostic evaluation, such as a biopsy, to confirm the presence of recurrent cancer, while scores of 1 or 2 may suggest a benign post-treatment course, often with routine follow-up. A score of 3 signals indeterminate results, and clinicians may opt for additional imaging to clarify the diagnosis [77].

### 2.2. Clinical Application

By calculating the TARGET score through this combined MRI assessment, radiologists and clinicians can more accurately determine recurrence likelihood. This process helps reduce under- and overtreatment, allowing for tailored patient management based on the specific characteristics of the treated prostate tissue.

#### 2.2.1. Background, Principles, and Criteria of Scoring in the PI-FAB System

The PI-FAB system is a 3-point scale that was officially introduced in 2023 by researchers at University College London, and, just like TARGET (a 5-point scale), it is a scoring framework aimed at evaluating mpMRI results in patients who have undergone FT for PCa and was created to address limitations in existing systems like PI-RADS [76,83].

PI-FAB involves a 3-point scale for rating MRI sequences in sequential order: (1) DCE sequences; (2) DWI, split into the assessment of the high b-value sequence first and then the apparent diffusion coefficient map; and (3) T2-weighted imaging [83].

#### 2.2.2. Key PI-FAB Recommendations

Similar to TARGET, it is essential that a comparison with the pretreatment scan is available to help with the assessment. Experienced readers must read the MRI, and key information such as the date of FT, tumor burden, ablation modality, and post-FT PSA must be provided to help in the clinical management decisions according to the PI-FAB score. It is also recommended that the scan be performed 12 months after focal therapy if PSA falls below the baseline; otherwise, as early as 6 months [84,85]. In order to assign a PI-FAB score, it is preferred that the scan meet the PI-RADS v 2.1 minimum technical recommendations and exhibit adequate diagnostic quality by receiving a PI-QUAL score of at least 4 [83,86,87].

#### 2.2.3. Calculating the PI-FAB Score (Table 2) (Figure 3)

**Figure 3:**
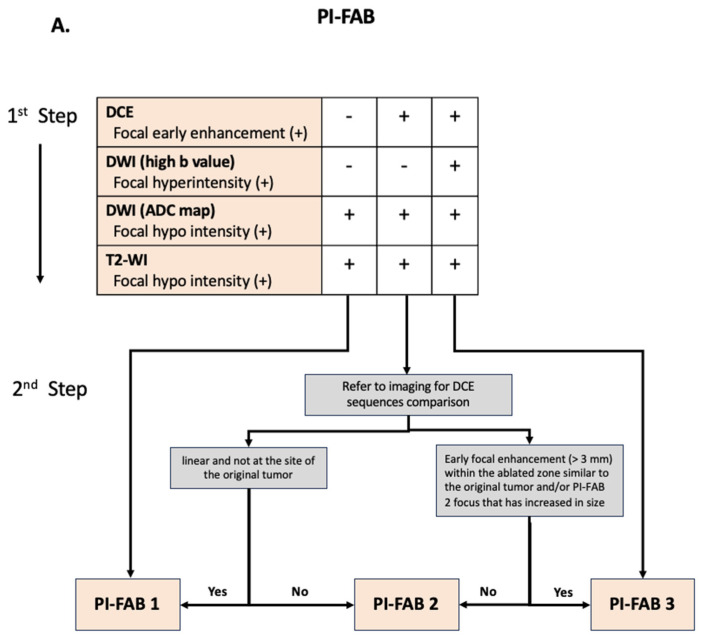

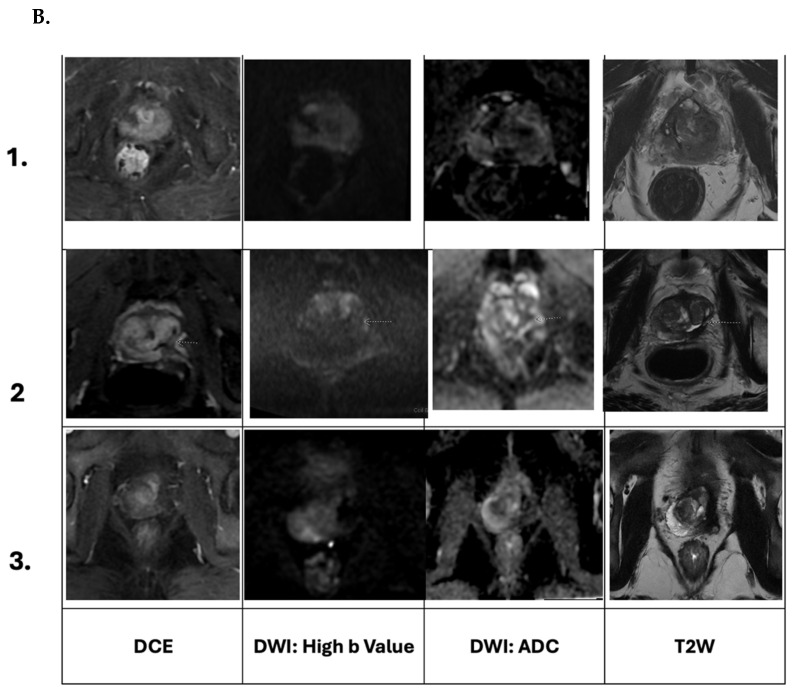
(**A**) Stepwise algorithm for characterizing lesions within the ablation zone following focal therapy based on a 3-point PI-FAB scoring system. In the first step, DCE is the most important sequence. In the second step, findings should be compared to the pre-focal therapy MRI to assess for a potential upgrade or downgrade of the PI-FAB score. (**B**) Examples of MRI scans after focal therapy, with varying findings within the ablation zone. Row 1: PI-FAB 3: MRI performed 12 months after right hemiablation with HIFU. At the anterior aspect near the apex, there is a focal lesion with early enhancement (DCE +), corresponding high-b images (DWI +), and restricted diffusion on ADC (+), as well as hypointensity on T2WI (+) corresponding to the prior lesion seen in prior exam. This would give an overall PI-FAB score of 3. Targeted biopsy of this lesion was positive for 7 of 12 cores, revealing grade group 3 cancer with a maximum cancer core length of 12 mm. Row 2: PI-FAB 2: MRI performed 9 months after right peripheral zone HIFU. At the posterolateral peripheral zone there is a focal lesion outlined by the dotted arrow in dynamic post contrast, DWI, ADC and T2W respectively showing mild enhancement in DCE (+), no signal in DWI (-), and positive hypointensity in ADC (+) and T2WI (+). This would give an overall PI-FAB score of 2. Targeted biopsy of this lesion was negative for malignancy. Row 3: PI-FAB 1: MRI performed 7 months after left-quadrant ablation with HIFU. There is no contrast enhancement within the ablation zone on DCE (-), T2 focal hypointensity, and diffusion restriction DWI (-), giving an overall PI-FAB score of 1. Targeted biopsies sampling the whole prostate were all negative. ADC= apparent diffusion coefficient; DCE = dynamic contrast-enhanced; DWI = diffusion-weighted imaging; HIFU = high-intensity focused ultrasound; T2W = T2-weighted.

PI-FAB employs a three-point scale to assess the three primary MRI sequences: DCE, DWI (split into the assessment of the high b-value sequence first and then the ADC map), and T2-WI in a structured sequence (Figure 3).

Figure 3 is the diagram used to indicate the likelihood of PCa recurrence utilizing the PI-FAB score and is summarized in Table 2. The PI-FAB score criteria are quoted below from Giganti et al. [83]:

“Low signal intensity on T2-WI and low signal intensity on high-b-value DWI, and no enhancement on DCE at the site of the original tumor:

PI-FAB 1: It likely represents fibrosis.

Focal enhancement alone (low signal intensity on T2-WI, low signal intensity on high-b-value sequence):

PI-FAB 1:

Findings: linear enhancing area and not at the site of the original tumor or the edge of the ablation cavity. It likely represents a vessel or inflammation.

PI-FAB 2:

Findings: Enhancing regions less than or equal to 3 mm and at the original tumor site.

PI-FAB 3:

Findings: Early focal enhancement of more than 3 mm within the ablated zone/edge of the ablation cavity or a PI-FAB 2 focus that has now increased in size.

High signal intensity on the high-b-value sequence and focal enhancement of any size and low signal intensity on T2-WI and on ADC map:

PI-FAB 3: high suspicion for residual or recurrent disease”.

Clinical Applications and Recommendations for Clinical Management:

Management decisions need to take into account the PI-FAB score from the MRI, PSA, risk stratification, and patient characteristics. Rather than solely using the PI-FAB score, the entire clinical picture should guide clinical management. When the patient and clinician agree to continue active management, the following recommendations should be considered based on the PI-FAB score [83]:

PI-FAB 1: Routine monitoring with mpMRI.

PI-FAB 2: Assess PSA kinetics—a rising PSA may necessitate biopsy. If the PSA is stable, delay biopsy and schedule MRI follow-up after one year.

PI-FAB 3: Prompt biopsy is recommended to evaluate recurrence.

## 3. Comparison of TARGET and PI-FAB

TARGET and PI-FAB were both developed to address the need for a standardized approach to interpreting post-FT mpMRI. The study by Esengur et al. (2024) [12] represents the first study to directly compare the diagnostic performance of these systems, providing initial insights into their relative strengths and limitations [74].

PI-FAB demonstrated consistently high sensitivity (92.9%) across both readers, suggesting potential reliability in detecting residual or recurrent disease. Its high negative predictive value (NPV) also appears promising for excluding clinically significant prostate cancer (csPCa) in cases with low scores. However, its moderate specificity (62.5% for reader 1 and 54.2% for reader 2) raises concerns about a tendency for false positives, which could lead to unnecessary follow-up procedures [74].

TARGET, on the other hand, showed higher specificity and positive predictive value (PPV), which may help reduce false positives and enhance diagnostic confidence. It also demonstrated slightly better diagnostic accuracy for both readers, with an overall accuracy of 78.9% for reader 1 and 73.7% for reader 2, compared to 73.7% and 63.4% for PI-FAB, respectively [74].

Despite these preliminary observations, the study highlighted several limitations that temper the interpretation of these results. Variability in reader performance, likely due to limited experience with both TARGET and PI-FAB, underscores the importance of standardized training to improve inter-reader reliability. Furthermore, the small sample size and inconsistencies in imaging protocols limit the generalizability of these findings. Larger studies with harmonized protocols are essential to validate these systems and better understand their clinical utility.

## 4. Limitations and Future Directions for TARGET and PI-FAB

Both TARGET and PI-FAB exhibit significant limitations that require attention for their broader clinical utility. Firstly, there are validation gaps, as neither scoring system has undergone large-scale, multi-institutional studies, which are critical for ensuring consistency and applicability across varied populations and clinical settings [80]. Secondly, both systems face challenges related to inter-reader variability [88,89,90]. This issue is particularly evident in PI-FAB, where differences in radiologists’ interpretations can lead to inconsistencies in scoring and subsequent management decisions [80,84]. Additionally, integration with clinical parameters remains limited. For instance, TARGET does not fully incorporate dynamic clinical factors like PSA kinetics, individualized patient histories, or emerging genomic markers, which restricts its predictive accuracy in certain cases [74]. Lastly, economic and resource challenges emerge as barriers, as advanced imaging modalities like mpMRI, which underpin these scoring systems, may be cost-prohibitive and inaccessible in low-resource settings [91,92,93,94,95].

Looking forward, several research opportunities can enhance the clinical utility of these scoring systems [96]. One promising avenue is the development of artificial intelligence-based web applications, leveraging deep machine learning to mitigate inter-reader variability and improve diagnostic accuracy for clinically significant recurrences [97,98,99,100]. Another critical area is biopsy correlation, where prospective studies aligning these scoring systems with histopathological findings from targeted biopsies could validate their relevance further [74,101]. Additionally, longitudinal outcome studies are essential to evaluate how TARGET and PI-FAB scores influence long-term treatment decisions and patient outcomes, which could refine their practical application [74]. Finally, there is a need to broaden their application through evaluation in diverse healthcare settings and integration into multidisciplinary care teams, ensuring equitable access and optimal management of post-focal therapy prostate cancer. These directions promise to bridge the current gaps and enhance the role of TARGET and PI-FAB in clinical practice.

## 5. Conclusions

The TARGET and PI-FAB scoring systems were introduced to assess prostate MRI after FT after recognizing the limitations of the existing PI-RADS for post-therapy imaging. These systems were specifically designed to account for the changes observed in MRI post-FT, including scar tissue, inflammation, and other treatment-related effects, to help radiologists differentiate between benign post-treatment changes and residual or recurrent cancer.

Given the recent development of the PI-FAB and TARGET scoring systems, research on their effectiveness is limited. Despite these challenges, the potential of PI-FAB and TARGET to enhance post-focal therapy evaluation and guide management decisions is significant, marking them as promising tools in prostate cancer care. Standardizing imaging assessments with TARGET and PI-FAB can improve diagnostic accuracy, enhance follow-up care, and aid clinical decision-making for patients undergoing FT.

## Figures and Tables

**Table 1:** TARGET score: calculation and clinical criteria and application.

Component	Details
Major Sequence	DCE-MRI (dynamic contrast-enhanced MRI) is the principal imaging for TARGET. Highlights vascular changes to differentiate benign from suspicious tissue post-therapy.
Minor Sequences	DWI (diffusion-weighted imaging) helps identify restricted diffusion (cancer suspicion). T2WI (T2-weighted imaging) assesses tissue morphology and scarring.
In the TARGET scoring system, lesions within the ablation zone are evaluated on a 5-point scale that reflects the level of suspicion for recurrence. Each sequence, DCE-MRI, DWI, and T2WI, is rated on a scale of 1 to 3, with individual sequence scores indicating the following:	
Individual Sequence Scoring	1 = Nonsuspicious 2 = Equivocal 3 = Suspicious
Combined TARGET Score (1–5)	1–2 (Low likelihood of recurrence): Benign features across all sequences. Score 1 for very low suspicion and score 2 for low suspicion. 3 (Indeterminate): Some suspicious features; requires additional imaging or follow-up. 4–5 (High likelihood of recurrence): Suspicious across sequences; DCE-MRI often decisive. Score 4 for high suspicion and score 5 for very high suspicion.
Clinical Application	Score 1–2: Routine follow-up with imaging. Score 3: Additional imaging recommended. Score 4–5: Recommend biopsy to confirm recurrence.

**Table 2:** **PI-FAB score: calculation and clinical criteria and application.** The PI-FAB scoring system uses MRI features to assess the likelihood of prostate cancer recurrence following focal therapy. Below is a summary table of the PI-FAB scores, including MRI findings, their interpretations, and clinical management recommendations.

PI-FAB Score	MRI Findings	Interpretation	Clinical Management
1	Low signal on T2-WI and Low signal on high-b-value DWI and No enhancement on DCE at the site of original tumor	Likely represents fibrosis	Routine monitoring with mpMRI
1	• Focal enhancement alone as - Low signal on T2-WI - Low signal on high-b-value DWI - Linear enhancing area and not at the site of the original tumor or the edge of the ablation cavity	Likely a vessel or inflammation	Routine monitoring with mpMRI
2	Enhancing region ≤ 3 mm and at the original tumor site	Suspicious, needs close follow-up	Assess PSA kinetics: if rising, biopsy; if stable, follow-up MRI after 1 year and delay biopsy
3	Early focal enhancement > 3 mm within the ablated zone or edge or a PI-FAB 2 focus that has now increased in size High signal on high-b-value DWI and focal enhancement of any size and low signal intensity on T2-WI and on ADC map	High suspicion for residual or recurrent disease	Prompt biopsy recommended

## Abbreviations

The following abbreviations are used in this manuscript:

DCE-MRI	Dynamic contrast-enhanced MRI
DWI	Diffusion-weighted imaging
FT	Focal therapy
HIFU	High-intensity focused ultrasound
IRE	Irreversible electroporation
mpMRI	Multiparametric magnetic resonance imaging
PCa	Prostate cancer
PI-FAB	Prostate Imaging after Focal Ablation
PI-RADS	Prostate Imaging Reporting and Data System
PI-RR	Prostate Imaging for Recurrence Reporting
PSA	Prostate-specific antigen
T2WI	T2-weighted imaging
TARGET	Transatlantic Recommendations for Prostate Gland Evaluation with MRI after Focal Therapy
